# Respectfully Submitted

**Published:** 1903-12

**Authors:** 


### TEXAS MEDICAL JOURNAL.

AUSTIN, TEXAS.

A MONTHLY JOURNAL OF MEDICINE AND SURGERY.

EDITED AND PUBLISHED BY
F. E. DANIEL, M. D.

ASSOCIATE EDITORS:

WITTEN BOOTH RUSS, M. D., San Antonio, Texas.

P. M. PAYNE, M. D., Brownwood, Texas.

Published Monthly at Austin, Texas. Subscription price $1.00 a year in advance.

Eastern Representative: John Guy Monihan, St. Paul Building, 220 Broadway, New York City.

Official organ of the the West Texas Medical Association, the Houston District Medical Association, the Austin District Medical Society, the Brazos Valley Medical Association, the Galveston County Medical Society, and several others.

THE "Red-Back" sends greetings to its thousands of friends everywhere, and wishes them all and singular, a merry Christmas and a happy and prosperous new year.

### RESPECTFULLY SUBMITTED.

"The action of the mosquito in transmitting the disease is not a simple inoculation from the sick to the well. In the first place, the mosquito can only become infected by biting the sick during the first three and a half days of the disease, and after the mosquito has become infected a period of from twelve to eighteen days must lapse before the mosquito can transmit the infection, and not exceeding six days thereafter the fever will develop in the person bitten."—*Dr. G. M. Guiteras, Surgeon Marine Hospital Service.*

Twelve to eighteen days, and (not over) six days incubation: Fifteen to twenty-four days via the mosquito route.

"L. M. W. went from Clinton to C.'s funeral, at Edwards, Miss., September 7, 1897; arrived about 11:30 a. m., and left that afternoon. She developed yellow fever at Clinton, September 10th, at night.

"M. C. went to C.'s funeral at Edwards, Miss., September 7th, 11:30 a. m., and left that afternoon for Nitta Yuma, Miss., and developed yellow fever September 11th, night."—*Dr. H. R. Carter, Surgeon Marine Hospital Service, in Medical Record, March 9, 1901.*

Dr. Jacob S. West, of Texas, is quoted as reporting two instances where coffee introduced infection. Both occurred in 1867, and are related as follows:

"At Liberty, Texas, a sack of coffee landed two miles below town from the steamboat Ruthven, which coming from Galveston, was refused permission to land at the wharf. This sack was taken on a dray to Liberty. All who shared the coffee were stricken with fever, which spread with disastrous effects."

"The second case was that of a sack of coffee which was hauled fifteen miles in an open wagon from Corpus Christi, where fever prevailed, to a point near Meansville, where it was divided among the purchasers. No one of them escaped and many of them died; but those who did not so share, singularly enough, escaped."

Keating reports on the source of infection at Covington, Tenn., as follows:

"The only person who had the fever at Covington, Tenn., in 1878, was the postmaster, who received and opened a heavy mail that had been detained in the Memphis postoffice for some time. In three days he died of yellow fever.

[See also Dr. McDaniel's letter in this issue.—EDITOR.]

The following lengthy extract is taken from Dr. J. H. Purnell's article in the *Philadelphia Medical Journal,* August 3, 1901: "The Mosquito an Insignificant Factor in the Propagation of Yellow Fever."

"Ordinarily, in mid-summer, we can control an epidemic if the people can be moved to a camp; Fortress Monroe, 1899, for example. As to the soiled material which was introduced into the little cabin [Reed and Carroll's experiment in Cuba] being sufficient upon which to base an absolute conclusion, is concerned, I point to the fact that in nearly every epidemic analagous instances may be found—La Roche teems with them—and cases of fever do not occur simply because the *infection* is absent. Stench and filth are incapable of producing infection. What does, is shrouded in mystery. The germs, as they are discharged from the human body, do not at first seem capable of infecting, but must remain for a time in an undisturbed atmospheric area which is suitable to their evolution before infection is possible. Unless the proper meteorological conditions prevail, the poison seems to be inert.

An illustration of the conditions not being present, is found in Keating's History of the Epidemic of 1878, in speaking of Camp Joe Williams, which was located near Memphis, and was peopled by a thousand or more refugees from the city. A number of persons who had become infected before leaving the city developed the disease after reaching the camp, but from such cases there was no spread. Surgeon R. B. Nall, who was in charge of the camp, says: "The remarkable and favorable feature of Camp Joe Williams, was that the disease did not spread amongst its inhabitants, nor did those who visited the camp from the surrounding country contract the disease. * * *

* * * "On the arrival of the refugees, every article of bedding and clothing which favored propagation of the disease was destroyed by fire. * * *

* * * "Five of the eight male nurses employed in the hospital, after nursing for three or four weeks fifteen or twenty patients in all stages of the fever. thinking themselves proof against the disease, went to the city to offer themselves to the Howard Association as nurses because of the higher prices paid. They found the sick bountifully supplied with nurses from elsewhere, and were unable to obtain positions, and returned to camp. Four of these men died in the hospital in which they had nursed—the fifth was found dead between the city and camp. The other three nurses did not visit the city, but remained in the hospital during the epidemic (seventy-two days), nursed and buried their comrades, but were not attacked themselves."

The foregoing is a plain, unembellished statement of a reputable physician, and contains several points of interest in connection with the recent investigations carried on in Cuba.

First. If the bedding and clothing in the hospital at Camp Joe Williams during the *summer* weather did not become infected, is there not good reason to suppose that the material taken from Las Animas Hospital and Quemados in November and December escaped likewise? The non-immune physicians and nurses who attended yellow fever patients in Las Animas in 1899 failed to develop the disease while there, but did develop it afterwards from an infected center. (Information from Surgeon H. R. Carter.)

Second. All bedding and clothing coming from the infected city was burned, and while yellow fever cases developed in those who had become infected before leaving the city, none occurred in any other—is it not reasonable to suppose that the precautionary measures were responsible for the immunity?

Third. Since it is known that mosquitoes plentifully abound around the suburbs, as well as in the city of Memphis, and as no measures were adopted looking to the destruction of the pests, is it reasonable to suppose they would have confined their stings to the inhabitants of the city and permitted the camp to remain unmolested?

The above cases are cited to show that yellow fever may, and does occur after exposure to the infection within less than the time required by the mosquito route, and the mosquito is not the sole factor in spreading the disease. I could cite many hundreds. I have seen it myself. I understand that at the late meeting of the

A. P. H. A. at Washington, there were "some emphatic remarks" made because of the lack of a more general acceptance of the dictum that the mosquito is the sole agency in yellow fever epidemics. Those who have had much experience with the yellow fever do not accept it. It is not proven. They have seen the contrary demonstrated too often. Until the inconsistency above pointed out can be "explained," they will not be convinced; nor will it be safe to neglect to guard against the introduction of the disease by other agencies. Should it prove true that we have only the mosquito to deal with, it will be "a consummation devoutly to be wished," and no one would rejoice more than yours truly and the "Red-Back." It will then no longer be necessary to quarantine, or *to perforate letters* in quest of the pesky things, and disinfect them, for fear they may contain mosquitoes—as is now practiced by the Marine Hospital Service experts—who don't believe the infection can be carried in any other way than in a mosquito's abdomen. Until that time I must still beg leave to differ with Brother Paul.

